# The oncology day hospital in Spain: an updated analysis of Spanish Society of Medical Oncology (SEOM) looking forward

**DOI:** 10.1007/s12094-016-1610-1

**Published:** 2017-01-12

**Authors:** C. Jara, F. Ayala, J. A. Virizuela, Oncology Day Hospital Task Force

**Affiliations:** 1 Medical Oncology, Fundación Alcorcón University Hospital, Alcorcón, Spain; 2 Medical Oncology, Morales Meseguer University General Hospital, Murcia, Spain; 3 Medical Oncology, Virgen Macarena University Hospital, Seville, Spain

The day hospital is the unit of care where, under a specialist physician’s supervision or indication, patients undergoing complex diagnostic methods or treatment requiring hours of continuous medical and/or nursing care, but not hospital admission, are treated or receive care [1]. Day hospitals arose from the experience in hematology in the USA in the 1970s. Patients with leukemias and lymphomas needed complex treatments, in addition to transfusions, which entailed frequent care at the hospital. Hospitalization, with the implications it had for these patients’ quality of life and high associated costs, could be avoided with these new care units. The rationale for the monographic oncology day hospital (ODH) is that cancer patient care accounts for 80% of the activity at general day hospitals and calls for a well-defined diagnostic and therapeutic care; furthermore, unlike other specialties, cancer patients’ care demand is usually scheduled.

The ODH offers a broad spectrum of diagnostic and therapeutic procedures (Table 1), making it possible to improve the quality of life in patients with advanced disease—even combining the treatment of their illness while remaining professionally active in some cases, relieving the pressure of care on conventional hospital services and decreasing health-care expenditure, a growing concern. As a unit dedicated to medical treatment, the ODH will adapt to the characteristics of patients receiving care and the treatments they need. Consequently, it must have the appropriate equipment and facilities, depending on the service portfolio, and enable their activities to be scheduled so as to optimize the care they provide. The role of oncology nursing is key to achieving the aims of integral patient care at the ODH, including patient intake, information and education about treatments, and collaboration in clinical research. Part of these activities must be conducted in specific visits with the oncological nursing staff, which already exist in most Spanish ODH. Suiting patient needs will also entail changes in services as cancer diagnostics and treatments evolve.

**Table 1:** Day oncology hospital service portfolio

Administration of chemotherapy and targeted therapies
Medical consultation prior to treatment and assessment of chemotherapy-induced and targeted therapy-induced toxicity
Consultation for patients participating in clinical trials
Blood and urine collection for laboratory analyses
Hemotherapy
Transfusion of packed red blood cells
Transfusion of platelets
IV drug administration
Nursing care
Vascular catheter/Port-A-Cath care
Recovery after diagnostic imaging examination or radiotherapy
Guided biopsy
Percutaneous drainage
Cavity punction
Diagnostic or therapeutic paracentesis
Diagnostic or therapeutic thoracocentesis
Spinal tap
Direct line consultation with nursing staff

Reproduced with the authorization of the Spanish Society for Medical Oncology (SEOM)

The task force created for this purpose in 2015 by the Sociedad Española de Oncología Médica (Spanish Society for Medical Oncology) has assessed the current situation of ODH in Spain by means of a questionnaire to which 52 oncology services of the 141 invited to participate responded [2] and updated the previous survey (2004–2005) [3]. The survey considered 62% of the ODH to be monographic in comparison to 38% of the former survey, with more architectural resources and equipment (treatment chairs and beds available). The ODH is currently coordinated by a supervisor (54%) or the head of department (40%) and has extensive personnel (median of 51.5 nurses, 7 nurse aides, and 2 administrative assistants); more than 80% have an oncologist on duty in the afternoon. In short, ODHs have developed considerably in the last 10 years. Nevertheless, there is still room for improvement in certain aspects: despite the remarkable improvement in electronic global management systems at Spanish hospitals compared to the previous survey, up to 15% of the ODHs do not have electronic oncological prescribing, reaching the 30% of ODH in larger hospitals (in 2004–2005, 60% of the ODHs still had manual prescription). Electronic appointment systems were in place in 77 and 46% had an electronic patient identification system, although only 19% had a patient call display system in the waiting room. One aspect in which much remains to be done is in the area of quality control: only one-third of the ODHs have some kind of system to monitor appointment punctuality or delay in initiating treatments and only 30% have an automatic, triple-data capture barcode scanner (patient, nurse, and drug). Overall, 20% are quality standard certified, albeit this figure being even lower for smaller hospitals (11%).

Clinical research is part of clinical oncology and ODH’s daily activity. Of the patients cared for at the oncology services, 10–15% are participating in a clinical trial. It is therefore not surprising that, according to the survey, 67% of ODH have research nursing staff and that 88% have clinical research coordinators (commonly known as “data managers”).

As previously pointed out, one of the areas for improvement of ODHs in Spain is computerization, particularly as regards prescribing chemotherapy. Electronic prescription of the chemotherapy treatment order facilitates the prescription process, integrating all the often complex variables that come into play and finally contributes to enhancing patient safety. In addition to decreasing medical errors, it can also lower costs, shorten hospital stay, and promote compliance with clinical guidelines [4]. Other benefits include greater standardization of care, incorporating clinical decision processes into practice, better interdepartmental communication, and possibility to obtain data to quantify health-care practice for clinical research or to evaluate the quality of the processes [5]. Moreover, using an application for electronic chemotherapy prescription can offer highly useful tools, such as notifications of drug–drug interactions or allergies, warning about inappropriate dosing, dose limitations based on age or kidney function, or show reminders to administer other support treatments, among others. The ENEAS 2005 study highlights the importance of this facet by revealing that 37% of hospitalization-related adverse events in Spanish hospitals have to do with medication, and, of these, 56% can be considered avoidable [6]. The integration of pharmacy departments into the ODH care team is a key issue to achieving safe and efficient chemotherapy prescription. In recent years, the gradual introduction of oral anti-neoplastic treatments with the same risks as with intravenous administration has led to new needs surrounding safety and reconciling medications and patient information, which we believe make it mandatory to include these services also in secure electronic prescription systems and make coordination with the oncological pharmacist and the ODH nursing staff all the more necessary [7, 8].

Improving ODH care processes also means that activity and quality indicators must be developed to enable the evaluation and quantification of care activity. The SEOM Day Hospital Task Force has proposed the incorporation of a series of indicators related to care structure or activity, as listed in Table 2 [2]. In addition, in light of the lack of measurements of the quality of care, it proposes that a catalog of quality indicators be created and agreed upon. Once again, the use of computer systems that automatically elaborate these indicators based on the quantification of daily activity is fundamental to improve ODH management. This is also a necessary step toward implementing programs targeting patient safety and quality systems, which will probably be the next step in developing ODHs in our setting.

**Table 2:** Proposed indicators for oncological day hospitals

Structure indicators
Patient capacity (treatment chairs, beds)
Waiting room capacity
Accessibility for people with reduced mobility
Activity indicators
Rate of blood draws per day
Number of patients cared for per shift or day
Occupation rate
Number of first visits to nursing staff
Number of treatments prepared and administered
Number of telephone consultations with nursing staff
Emergencies in ODH
Number of patients referred to the emergency room or hospital admission
Mean treatment time per day or mean treatment duration based on complexity
Specific quality indicators
Cases of reactions to transfusion per 100 patients
Cases of adverse reactions to drugs or biological therapies
Nosocomial infection rate
PICC or central line complications
Extravasation rate in ≤5% of cycles
Accessibility indicators
Indicators of satisfaction of client expectations
Number of patient-proposed complaints and suggestions

As regards the future, the ODH not only comprises the critically important structure in outpatient cancer patient care, but also forms the centerpiece where most innovations in oncological treatment and the organization of cancer patient care converge. The incorporation of new technologies will enable this model to be expanded and transformed, yielding new opportunities to communicate with patients, monitor symptoms and treatment toxicity, and coordinate with other structures and levels of care. Telephone support for patients from the ODH, including care for oncological emergencies and monitoring toxicity [9], the use of e-mail in care processes, following existing recommendations to guarantee its proper use [10], and the introduction of specific mobile applications to monitor cancer patient’ symptoms or control toxicity [11–14], can undoubtedly enhance patient care in the ODH. Finally, important aspects to be developed in the ODH in the coming years would be coordination with primary care centers, their inclusion in the models of emergency care for patients with cancer, and the possible integration of palliative care within the structure of the ODH.

In conclusion, the members of the task force believe that the ODH should be the center of multidisciplinary care for cancer patients and that other models of care, more fragmented in the wake of the phasing out of the intravenous route of administration, do not offer the necessary safety or quality guarantees. The participation of clinical oncologists in their management, in close collaboration with the other professionals involved, is essential if the requirements as regards integral, high-quality and efficient care are to be met. In the coming years, we should witness the universal implementation of computerized prescription and management systems, quality and safety program implementation in the ODH, the introduction of new patient communication systems, and the organizational changes required to address the challenge posed by new cancer treatments. The introduction of accreditation programs, preferably driven from the SEOM in collaboration with other scientific societies, can be a key step toward achieving these aims and to assure adequate care for cancer patients.
